# CXCR3 predicts the prognosis of endometrial adenocarcinoma

**DOI:** 10.1186/s12920-023-01451-9

**Published:** 2023-02-07

**Authors:** He Dong, Mengzi Sun, Hua Li, Ying Yue

**Affiliations:** 1 Department of Gynecologic Oncology, The First Hospital of Jilin University, Changchun, China; 2 Department of Epidemiology and Statistics, School of Public Health, Jilin University, Changchun, China; 3 Department of Abdominal Ultrasound, The First Hospital of Jilin University, Changchun, China

**Keywords:** Endometrial adenocarcinoma, Immune, Gene marker, Prognosis, Nomogram

## Abstract

**Objectives:**

Currently, endometrial adenocarcinoma lacks an effective prognostic indicator. This study was to develop and validate a gene biomarker and a nomogram to predict the survival of endometrial adenocarcinoma, explore potential mechanisms and select sensitive drugs.

**Methods:**

425 endometrial adenocarcinoma cases with RNA sequencing data from TCGA were used to identify the most immune-related module by WGCNA. As an external test set, 103 cases from GSE17025 were used. Immune-related genes were downloaded from Innate DB. The three sets of data were used to identify the prognostic genes. Based on 397 cases with complete clinical data from TCGA, randomly divided into the training set (n = 199) and test set (n = 198), we identified CXCR3 as the prognostic gene biomarker. Age, grade, FIGO stage, and risk were used to develop and validate a predictive nomogram. AUC, C-index, calibration curve and K–M estimate evaluated the model's predictive performance. KEGG enrichment analysis, immune functions, TMB, the effectiveness of immunotherapy, and drug sensitivity between the high-risk and low-risk groups.

**Results:**

CXCR3 was identified as a prognostic biomarker. We calculated the risk score and divided the cases into the high-risk and low-risk groups by the median value of the risk score. The OS of the high-risk group was better than the low-risk group. The risk was the prognostic indicator independent of age, grade, and FIGO stage. We constructed the nomogram including age, grade, FIGO stage, and risk to predict the prognosis of endometrial adenocarcinoma. The top five KEGG pathways enriched by the DEGs between the high- and low-risk groups were viral protein interaction with cytokine and cytokine receptors, cytokine-cytokine receptor interaction, chemokine signaling pathway, natural killer cell-mediated cytotoxicity, and cell adhesion molecules. We analyzed the difference in immune cells and found that CD8+ T cells, activated CD4+ T cells, T helper cells, monocytes, and M1 macrophages were infiltrated more in the low-risk group. However, M0 macrophages and activated dendritic cells were more in the high-risk group. The immune function including APC coinhibition, APC costimulation, CCR, checkpoint, cytolytic activity, HLA, inflammation-promoting, MHC-I, parainflammation, T cell coinhibition, T cell costimulation, type I-IFN-response, and type II-IFN-response were better in the low-risk group. TMB and TIDE scores were both better in the low-risk group. By ‘the pRRophetic’ package, we found 56 sensitive drugs for different risk groups.

**Conclusion:**

We identified CXCR3 as the prognostic biomarker. We also developed and validated a predictive nomogram model combining CXCR3, age, histological grade, and FIGO stage for endometrial adenocarcinoma, which could help explore the precise treatment.

**Supplementary Information:**

The online version contains supplementary material available at 10.1186/s12920-023-01451-9.

## Introduction

Endometrial adenocarcinoma is the most common pathological type of endometrial cancer, which is the most common gynecological cancer in the United States. Since the mid-1990s, the mortality and related mortality of uterine corpus cancer has increased [1]. The disease was frequently symptomatic at an early stage, and endometrial cancer is often diagnosed at stage I [2]. Surgery is the preferred treatment for endometrial cancer, and the staging is based on the pathological evaluation after surgery [3]. Immunotherapy has a considerable clinical response in some relapsed or refractory cases [4–6]. With the continuous elucidation of the pathogenesis of endometrial cancer, more and more evidence indicate that many immune cells and cytokines can be seen in endometrial cancer tissue and stimulate an endogenous anti-tumor immune response. Compared with other gynecological malignancies, endometrial cancer is most likely to benefit from immunotherapy [7–9].

An integrated genomic analysis by The Cancer Genome Atlas (TCGA) resulted in the molecular classification of endometrioid and serous carcinomas into four distinct subgroups, POLE (ultramutated), microsatellite instability (hypermutated), copy number low (endometrioid), and copy number high (serous-like) [10]. In March 2020, the National Comprehensive Cancer Network (NCCN) recommended the molecular typing of the Cancer Genome Map Research Network for endometrial cancer for the first time. The main cancer immunotherapy methods include Immune checkpoint inhibitors, cancer vaccines, adoptive cell transfer, and lymphocyte-promoting cytokines [11]. Compared with TCGA molecular classification, gene testing is required to determine POLE status first, which is expensive. ProMisE, another commonly used molecular typing, is a simplified version. The typing first determines DNA MMR status and then determines POLE and P53 status, divided into POLE mutant, MMRd type, P53wt normal/wild type, and P53aba abnormal/mutant type [12, 13]. The relatively low cost is the advantage of using this typing to guide treatment. Combined with PD-L1 and TMB-H, molecular typing was used to help evaluate choosing immunotherapy, but it cannot be decided. Several immune-related biomarkers exist [14–18], consisting of multiple genes in endometrial cancer. Therefore, it will be more appliable and convenient to develop a simple prognostic biomarker consisting of fewer genes in endometrial adenocarcinoma for helping clinical practice. TCGA [19, 20] and Gene Expression Omnibus (GEO) [21, 22] have become popular sources of gene databases. Bioinformatics tools, such as weighted gene co-expression network analysis (WGCNA) [23], least absolute shrinkage and selection operator (LASSO) [24], and TIDE (Tumor Immune Dysfunction and Exclusion) algorithm (http://tide.dfci.harvard.edu) have been used to process data. Combining bioinformatics tools and these databases in the scientific study is reliably supported [25–29]. RNA sequencing (RNA-seq) data has the advantage of direct sequencing, a large amount of data, species restriction, and high data flexibility over expression array and NanoString. As RNA-seq data was more suitable for biomarker identification, we analyzed RNA-seq data and clinical data from databases with bioinformatics tools to identify an immune-related prognostic biomarker including only one gene, CXCR3, and develop a predictive nomogram for only endometrial adenocarcinoma to predict the prognosis and help select the sensitive drugs.

## Materials and methods

### Data collection and differentially expressed genes (DEGs)

Four hundred twenty-five endometrial adenocarcinoma cases with RNA-seq data from TCGA were used to identify the most immune-related module by WGCNA. (Additional file 1: Table S1). The immune score, stromal score, and estimated score for each patient were calculated by ESTIMATE [30], and 5559 immune-related genes (IRGs) were downloaded from InnateDB [31] (Additional file 1: Table S1). One hundred three cases with RNA-seq data from GSE17025 [21, 22] were used as an external test, and their DEGs were processed by GEO2R (Additional file 1: Table S1). The above three sets were used to identify and validate the prognostic genes to predict the prognosis of endometrial adenocarcinoma.

Three hundred ninety-seven endometrial adenocarcinoma cases from TCGA had complete clinical data, including age, histological grade, FIGO stage, vital status, time to death, and time to the last follow-up. These cases were randomly divided into a training set (n = 199) and a test set (n = 198) to develop and validate the risk model and predictive nomogram.

### WGCNA

WGCNA is a proper bioinformatics method for exploring immune-related modules. We first removed outlier genes and genes with extremely low expression from the data. A weighted gene network was constructed to raise co-expression similarity to calculate adjacency by choosing the soft thresholding power β. 22 was chosen as the soft threshold based on the approximate scale-free topology. We calculated adjacency and generated a hierarchical clustering tree. 11 modules with similar expression profiles were identified by dynamic tree cutting. Modules with highly co-expressed genes were merged. Finally, we associated the modules with the immune traits (i.e., immune score, stromal score, and estimate score) and chose the most relevant module.

### Identification and validation of the most immune-related gene biomarker

The overlapping genes were intersected by the most relevant module of WGCNA (module dark-orange), DEGs of GSE17025, and IRGs from InnateDB. Univariate Cox regression analysis, LASSO regression analysis, and multivariate Cox regression analysis were successively used to obtain the prognostic gene biomarker on the training set and tested by the test set. The risk score was calculated by multivariate Cox regression analysis. The risk score formula was: Risk score = ∑^n^_i = 1_Coefi × Expi. In this formula, Coefi is the coefficient of the prognosis-related gene, and Exp is the expression level of each retained gene. The median value of the risk score divided the cases into high- and low-risk groups. The predictive performance of the gene biomarker was evaluated by the area under the curve (AUC), concordance index (C-index), and Kaplan–Meier (K–M) estimate. The theoretical value of the C-index is between 0 and 1. If the C-index exceeds 0.5, the prediction performance is better than a random guess. Independent prognostic analysis can determine whether our model can be used as an independent prognostic factor independent of other clinical factors.

### Database for annotation, visualization, and integrated discovery (DAVID)

DAVID (version 6.8) [32, 33] is an online annotation tool used to interpret the biological function of gene sets. Here, we used the gene set of the most relevant module from WGCNA to do the Kyoto Encyclopedia of Genes and Genomes (KEGG) Pathway and Gene Ontology (GO) analysis by DAVID. GO analysis can provide biological information on gene function. KEGG pathway analysis can indicate the possible signaling pathways of the gene set. We input gene symbols onto the website and chose Homo Sapiens as the species. GO-BP-direct, Go-CC-Direct, Go-MF-Direct, and KEGG PATHWAY were selected for functional annotation. Other parameters were set to default.

### Comprehensive analysis of protein–protein interaction (PPI) network

PPI was performed by STRING [34], a Search Tool for the Retrieval of Interacting Genes Database (https://www.string-db.org/). The website analyzed overlapping genes to explore the possible relationships between them.

### Evaluating the difference in immune function and immune cells between the high- and low-risk group

Based on the 'limma' [35], 'GSVA' [36], 'GSEABase' [37], ‘ggpubr’ [38], and 'reshape2' [39] R packages, we calculated the different immune functions and cells between the high-risk and low-risk groups.

### Evaluating tumor mutation burden between the high- and low-risk group

TMB refers to the relative number of gene mutations in specific tumor tissue. Calculation formula: TMB (mut/Mb) = total mutation number (including synonymous, non-synonymous point mutation, replacement, insertion, and deletion mutation)/coding area size of a target area.

Perl (https://www.perl.org/) was used to extract somatic mutation information and estimate the TMB value. Then we used R to combine the patient's TMB information with clinical information, including survival time and status. The ' survminer ' [40]package was used to calculate the best cutoff value of TMB. According to the optimal critical value, the patients were divided into a high-TMB group and a low-TMB group. K–M survival analysis and the log-rank tests were performed to compare the OS differences between the above two TMB groups, and the OS differences in the four TMB groups combined with the risk score were compared. In addition, we explored the difference in TMB between the high-risk group and the low-risk group.

### Drug sensitivity and immunotherapy

We estimated the half-maximal inhibitory concentration (IC50) of commonly used chemotherapy drugs via the 'pRRophetic' [41] package. The potential response to immunotherapy was predicted and verified by the TIDE algorithm (http://tide.dfci.harvard.edu). The lower the TIDE score is, the more sensitive to immune checkpoint blockade (ICB).

### Development and validation of a nomogram

To explore the prognostic significance of gene biomarkers and clinical traits (such as age, histological grade, and FIGO stage), we developed a predictive nomogram to evaluate the prognosis of endometrial adenocarcinoma. First, the cases were grouped by the median predicted risk score, and the survival differences between the two groups were compared by K–M plot and log-rank test. Second, calibration curves evaluated the consistency between the predicted and the actual survival probability at 3 and 5 years. A 45-degree calibration curve indicates a perfect prediction.

### RNA extraction and quantitative RT-PCR

Trans Script All-in-One First-Strand cDNA Synthesis SuperMix for qPCR (One-Step gDNA Removal) by TransGen Biotech (Beijing, China) was used to extract total RNA from tissues. Reverse transcription of total RNA into cDNA was performed with EasyPure RNA Kit by TransGen Biotech (Beijing, China) for real-time PCR analysis. Specific PCR primers were designed by Comate Bioscience Co., LTD. (Jilin, China). The fold change in expression was calculated using the 2-∆∆Ct method, with GAPDH as an internal control. The primer sequences: GAPDH: Forward: AATTCCATGGCACCGTCAAG, Reverse: AGCATCGCCCCACTTGATTT; CXCR3: Forward: TACTGCTATGCCCACATCCTG, Reverse: TGATAGGGGGTCCAGCAGAG.

### Statistical analyses

All statistical analysis was performed in the R Studio software (version 3.6.1). R packages such as ‘caret’ [42], ‘dplyr’ [43], ‘WGCNA’ [23, 44], ‘limma’ [35], ‘GSVA’ [36], ‘GSEABase’ [37], ‘ggpubr’ [38], and ‘reshape2’ [39], ‘pheatmap’ [45], ‘pec’ [46], ‘regplot’ [47], ‘stringr’ [48], ‘flashClust’ [44], ‘glmnet’ [49, 50], ‘ggplot2’ [51], ‘org.Hs.eg.db’ [52], ‘DOSE’ [53], ‘enrichplot’ [54], ‘survival’ [55, 56], ‘survminer’ [40], ‘timeROC’ [57], ‘rms’ [58], ‘circlize’ [59], ‘RColorBrewer’ [60], ‘ComplexHeatmap’ [61],’ maftools’ [62], ‘clusterProfiler’ [63, 64], and 'pRRophetic' [41] were used. Continuous variables between the two groups were analyzed using a t-test. The Wilcoxon test performed a non-parametric comparison between the two groups. *P* < 0.05 was considered statistically significant.

## Result

### Study protocol

**Fig. 1:**
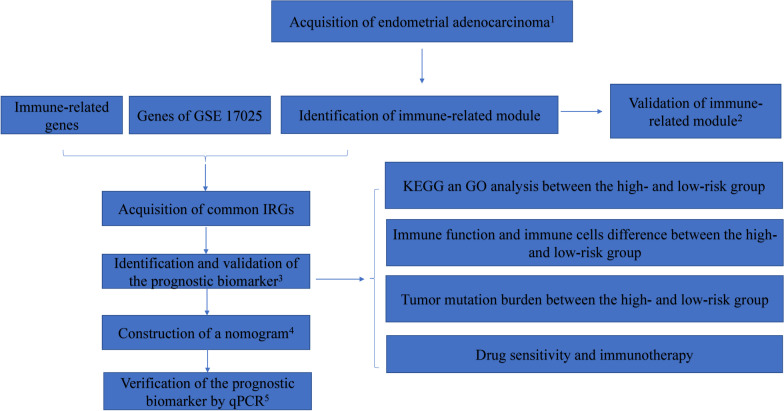
Flow chart depicting the protocol. ^1^Four hundred twenty-five endometrial adenocarcinoma cases with RNA-seq data from TCGA were used to analyze the most immune-related genes. One hundred three endometrial adenocarcinoma cases with RNA-seq data from GSE17025 were used as an external test set. Moreover, 5559 immune-related genes were downloaded from InnateDB. The three sets were used to identify and validate prognostic gene biomarkers. ^2^We used KEGG analysis and GO analysis to determine if the most relevant module correlated to immunity. ^3^The gene biomarker was identified and validated from 53 overlapping IRGs using univariate Cox regression analysis, LASSO regression analysis, and multivariate Cox regression analysis based on the training set (n = 199) and the test set (n = 198). Twenty-eight cases with missing clinical data were excluded from the study. ^4^Age, histological grade, FIGO stage, and risk group were used to develop and validate the prognostic nomogram. ^5^QPCR verified the relative expression between normal endometrium tissue and endometrial adenocarcinoma tissue

The schematic diagram of the study protocol is shown in Fig. 1.

### Identification of GSE17025 DEGs in endometrial adenocarcinoma

**Fig. 2:**
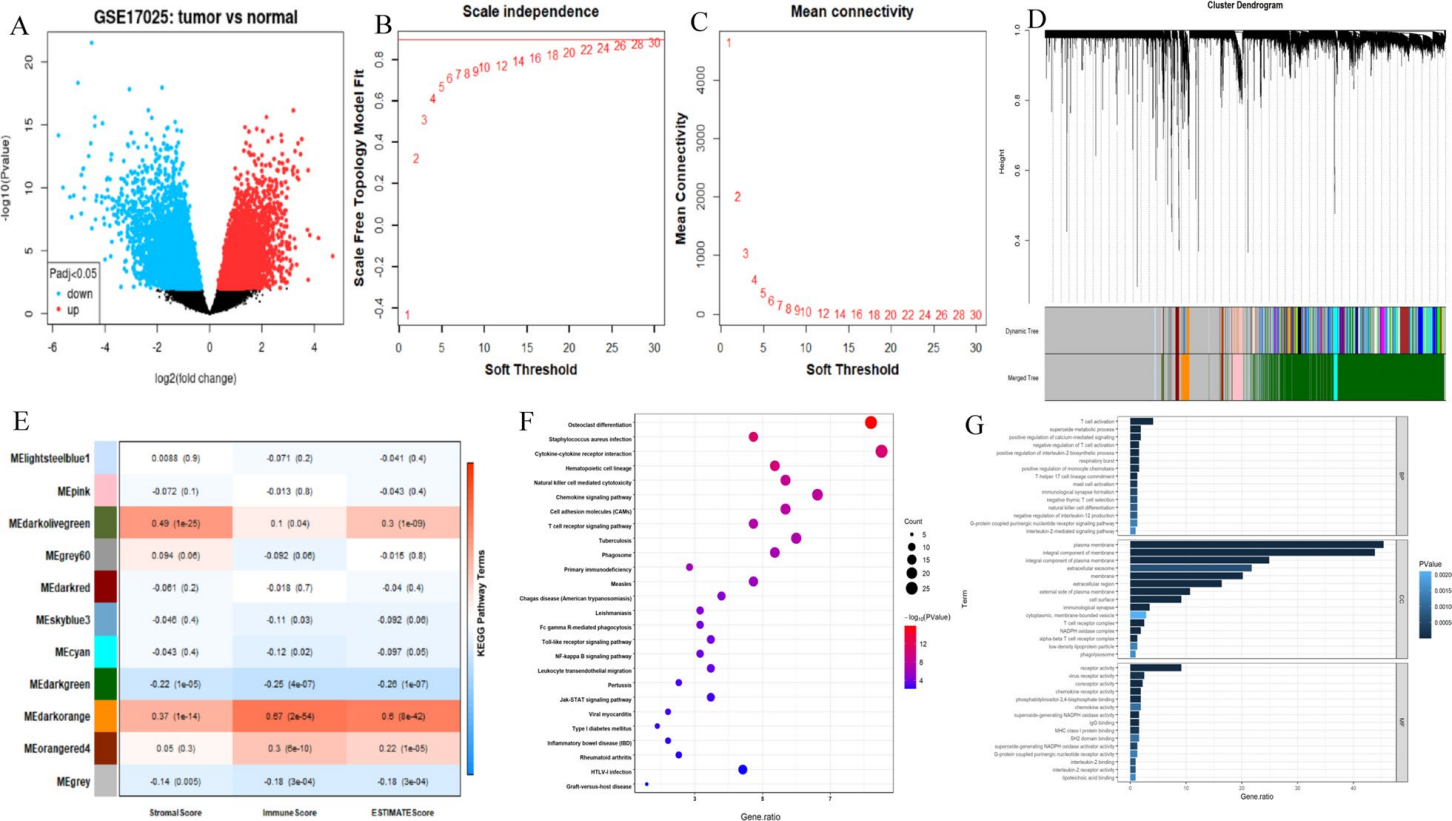
Identification and validation of immune-related modules **A**. Volcano plot of GSE17025 DEGs. **B**, **C** Determination of soft threshold power in WGCNA. **B** The panel showed a scale-free index for various soft-threshold powers (β). **C** This panel showed the mean connectivity for various soft-threshold powers. **D** Clustering dendrogram of genes, with dissimilarity based on the topological overlap, together with assigned module colors. **E** Module-trait association. Each row represents a module, and each column represents a feature. Each cell contains the corresponding correlation and *P* value. The dark-orange module (MEdarkorange) was most correlated with the immune score (*P* = 2e−54, r = 0.67). **F** Most of the KEGG pathways were also associated with immunity, demonstrating that the dark-orange module was immune-related. **G** Most of the categories in GO enrichment analysis based on genes in the dark-orange module were related to immunity, supporting that the dark-orange module was immune-related

One hundred three cases of endometrial adenocarcinoma from GSE17025 were used as an external gene set to develop the prognostic gene biomarker using RNA-seq data. 4697 DEGs were obtained by GEO2R. Adjust *P* value < 0.05 and | log FC |> 1 were set as the criteria. DEGs were displayed in the volcano plot (Fig. 2A).

### Identification and validation of the most immune-related module

The 425 cases with RNA-seq data and the corresponding immune score of each endometrial adenocarcinoma case were analyzed by WGCNA to identify the most immune-related module. 38,589 genes with extremely low expression levels were filtered out. 16,299 WGCNA candidate genes were obtained. 22 was set to be the soft threshold (Fig. 2B–C). All genes were classified and merged into the 11 highly co-expressed modules (Fig. 2D). Among them, the dark-orange module was the strongest immune-related module (*P* = 2e−54, r = 0.67). The module contained 325 genes. In addition, the dark-orange module was also significantly correlated to the stromal scores and estimate scores in endometrial adenocarcinoma (Fig. 2E). DAVID analyzed 325 genes in the dark-orange module to testify whether this module was immune-related. All pathways in GO and KEGG were found to be immune-related. (Fig. 2F–G).

### Identification of overlapping genes and PPI network

**Fig. 3:**
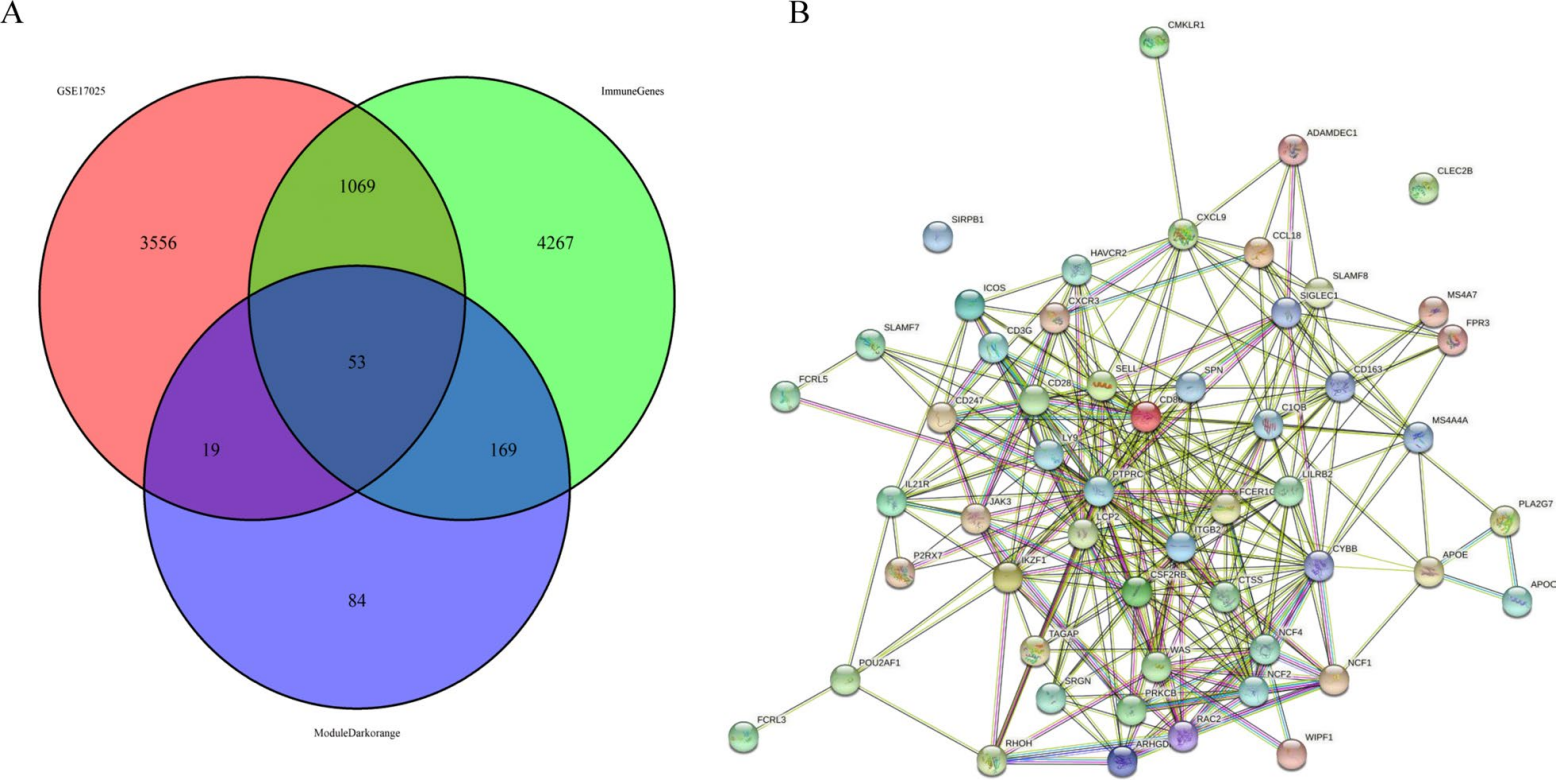
Identification of the overlapping genes by intersecting the genes from the dark-orange module, IRGs from InnateDB, and DEGs from GSE17025. **A** Venn diagram of GSE17025, the dark-orange module, and IRGs. **B** PPI network of overlapping genes

Fifty-three overlapping genes were obtained by intersecting 325 genes from Module dark-orange, 5559 IRGs from InnateDB, and 4697 DEGs from GSE17025 to select eligible immune genes to develop the immune-related gene biomarker (Additional file 1: Table S1, Fig. 3A). STRING was used to explore the interaction between overlapping genes (Fig. 3B).

### Identification and validation of prognostic immune-gene biomarker

**Fig. 4:**
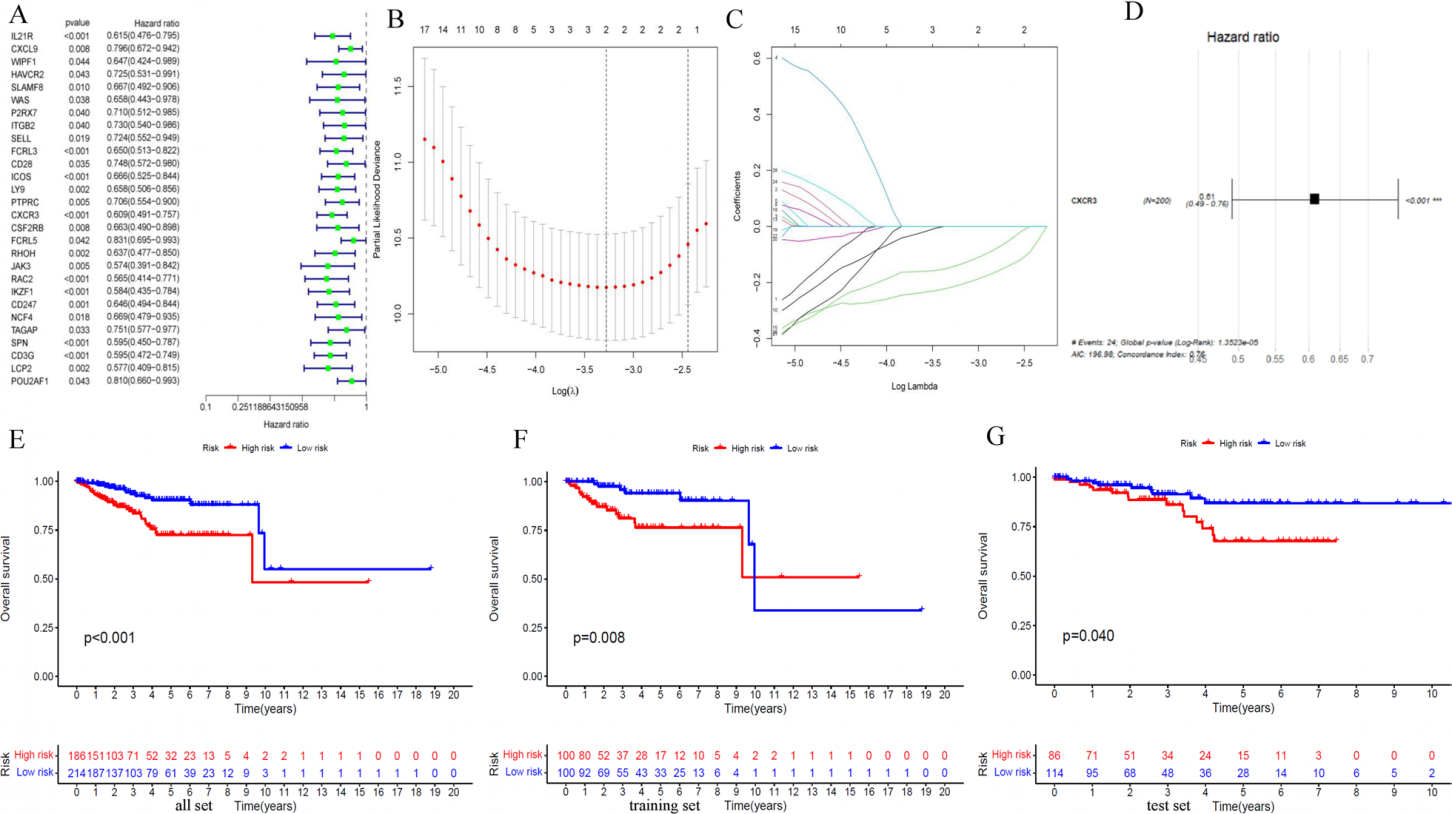
Identification and validation of the gene biomarker. **A**–**D** Univariate Cox regression analysis was used to identify the potential prognostic gene biomarkers. LASSO regression was used to eliminate redundant genes further. **A** Twenty-eight genes were screened to basing on univariate Cox regression analysis. **B** Tuning parameter (λ) selection in the LASSO model used tenfold cross-validation via minimum criteria. **C** LASSO coefficient profiles of 2 variables against the log (λ) sequence. **D** Multivariate Cox regression analysis was used to screen for genes significantly associated with overall survival. As shown in the forest plot, CXCR3 with a *P* value less than 0.05 was significantly associated with overall survival. **E–G** The survival difference between the low-risk and high-risk groups was divided by the median risk score threshold using multivariate Cox regression analysis. In the all-set, training, and test set, the survival of the low-risk group was significantly longer than that of the high-risk group

**Fig. 5:**
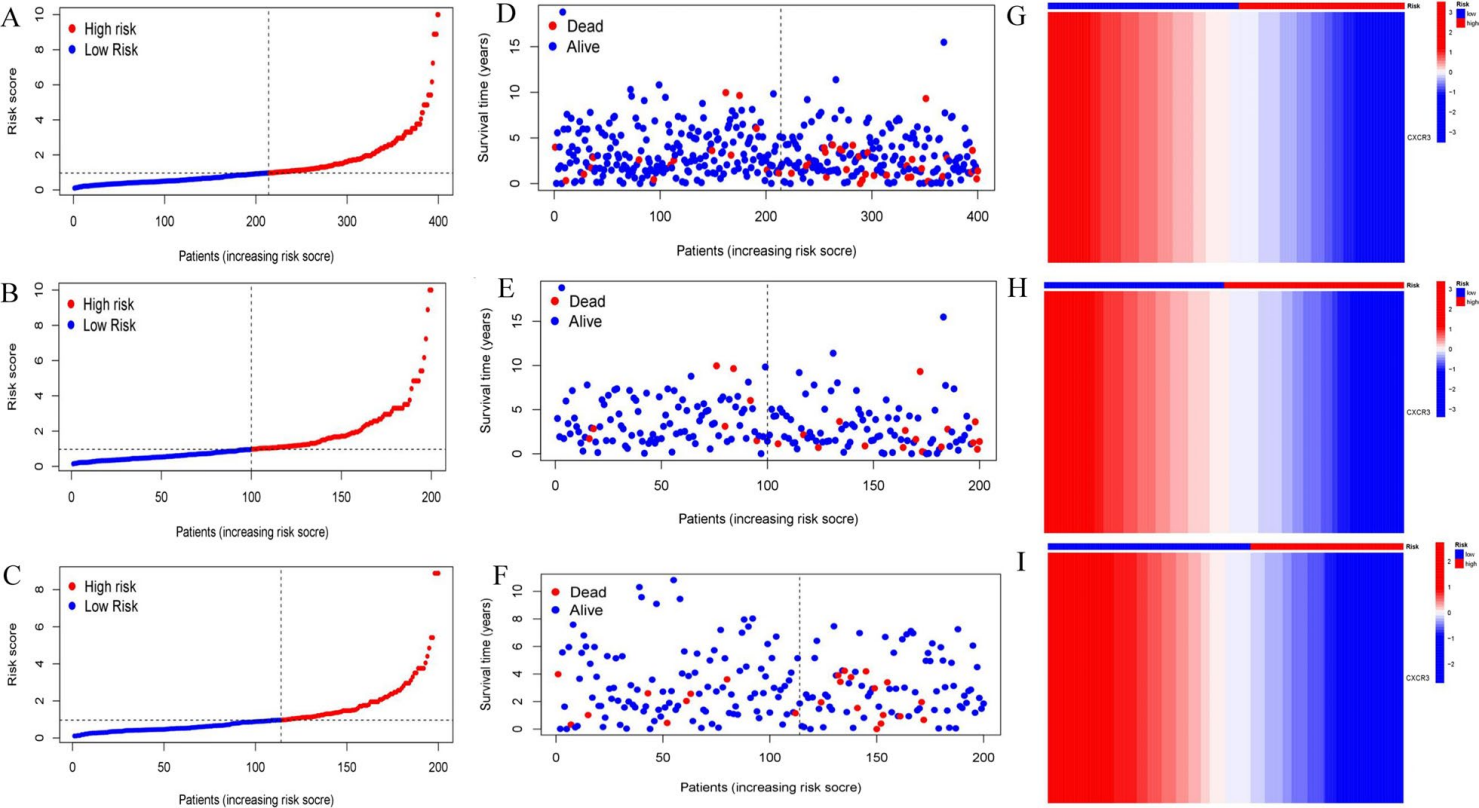
Risk curves. **A–C** The vertical axis represents patients' risk score, and the horizontal axis represents the number of patients. The patients were divided into high- and low-risk groups according to the median value of the risk score. The low-risk group was shown in blue, and the high-risk group was shown in red. **D**–**F** The vertical axis represented the survival time (unit: years), and the horizontal axis represented the number of patients. The red dot represented the dead patient, and the blue dot represented the living patient. As the risk increased, the number of dead patients increased. **G**–**I** It represented the difference in CXCR3 expression between high- and low-risk groups. The low-risk group had a high expression, and the high-risk group had a low expression, indicating that CXCR3 might be an anti-tumor gene

**Fig. 6:**
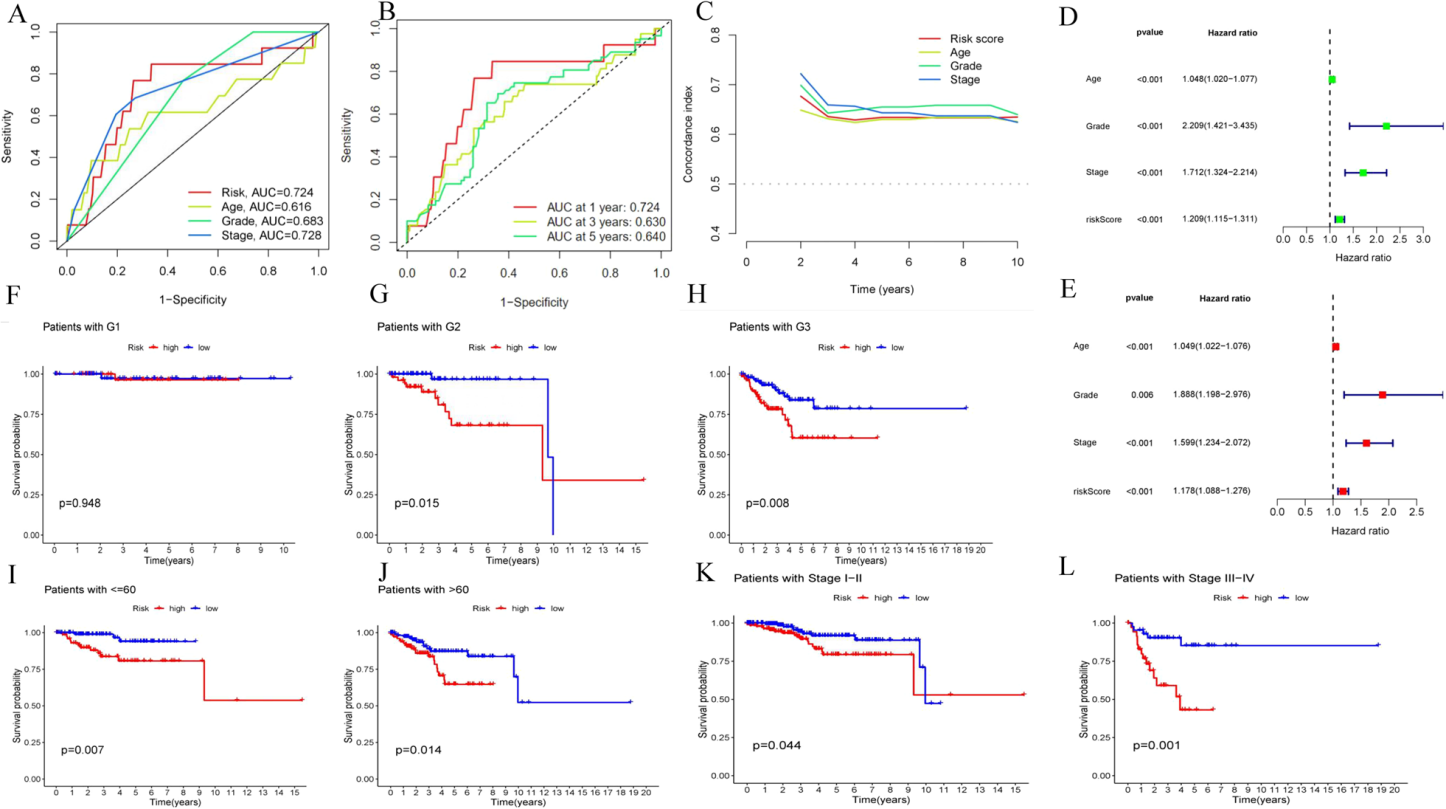
AUC and the independent prognostic analysis. **A–B** AUC and C-index were calculated for the gene marker. **A** AUC was calculated for the risk score, age, histological grade, and FIGO stage. **B** AUC was calculated at one year, three years, and five years. **C** The C-index of the risk, age, histological grade, and FIGO stage was calculated separately. **D–E** Univariate Cox regression analysis and multivariate Cox regression analysis were used to doing the independent prognostic analysis, which indicated that risk score was the prognostic marker independent of age, histological grade, and FIGO stage. **D** Univariate Cox regression analysis. **E** Multivariate Cox regression analysis. **F–L** Subgroup analysis of age, histological grade, and FIGO stage. **F** In patients of histological grade G1, the survival probability of the two risk groups had no difference (*P* = 0.948). **G–H** In patients of histological grades G2 and G3, the survival probability of the low-risk group was higher than the high-risk group (*P* = 0.015, *P* = 0.008 separately). **I–J** In patients younger than 60 years and elder than 60 years, the survival probability of the low-risk group was higher than the high-risk group (*P* = 0.007, *P* = 0.014 separately). **K–L** In patients of stage I–II and stage III–IV, the survival probability of the low-risk group was higher than the high-risk group (*P* = 0.044, *P* = 0.001 separately)

Three hundred ninety-seven endometrial adenocarcinoma cases with RNA-seq data and complete survival data from TCGA were randomly divided into a training set (n = 199) and a test set (n = 198) (Additional file 2: Table S2). Baseline characteristics were comparable between the sets (Table 1). First, 28 genes were selected from 53 overlapping genes based on the training set with univariate Cox regression analysis (*P* < 0.05) (Fig. 4A). Second, CXCR3, successively screened by LASSO regression and multivariate cox regression, was identified as the gene biomarker in the training set and validated in the test set (Fig. 4B–D). Third, based on the gene biomarker, we calculated the risk score of each case with multivariate Cox regression and then separated all the endometrial adenocarcinoma cases into the high-and low-risk group by the median value of the predicted risk score (the median value of risk score in the training set was 0.97) (Fig. 5A–J). The survival difference between the two groups was significant (*P* < 0.05; Fig. 4E–G). Fourth, the AUC of the risk, age, histological grade, and FIGO stage was 0.724, 0.616, 0.683, and 0.728, separately (Fig. 6A). The AUC for one year, three years, and five years was 0.724, 0.630, and 0.64, separately (Fig. 6B). The C-index of the risk score was larger than 0.5 (Fig. 6C). Fifth, independent prognostic analysis testified that risk score was a prognostic indicator independent of age, histological grade, and FIGO stage (Fig. 6D–E). In the subgroup analysis of patients by age, histological grade, and FIGO stage, we found the survival probability in the low-risk group was higher than the high-risk group in stratification such as histological grade of G2, G3, age more than 60 years, age less than 60 years, FIGO stage I–II and FIGO stage III–IV. For patients with a histological grade of G1, the survival probability between the two groups was no different (Fig. 6F–L).

**Table 1:** Baselisne of patients in the test set and the training set

Covariates	Type	Total	The test set	The training set	*P*-value
Age	< = 60	178 (44.5%)	88 (44%)	90 (45%)	0.9199
	> 60	222 (55.5%)	112 (56%)	110 (55%)	
Grade	G1	97 (24.25%)	45 (22.5%)	52 (26%)	0.474
	G2	117 (29.25%)	56 (28%)	61 (30.5%)	
	G3	186 (46.5%)	99 (49.5%)	87 (43.5%)	
Stage	Stage I	284 (71%)	147 (73.5%)	137 (68.5%)	0.0897
	Stage II	34 (8.5%)	21 (10.5%)	13 (6.5%)	
	Stage III	69 (17.25%)	26 (13%)	43 (21.5%)	
	Stage IV	13 (3.25%)	6 (3%)	7 (3.5%)

### Functional enrichment analysis between the high- and low-risk groups

**Fig. 7:**
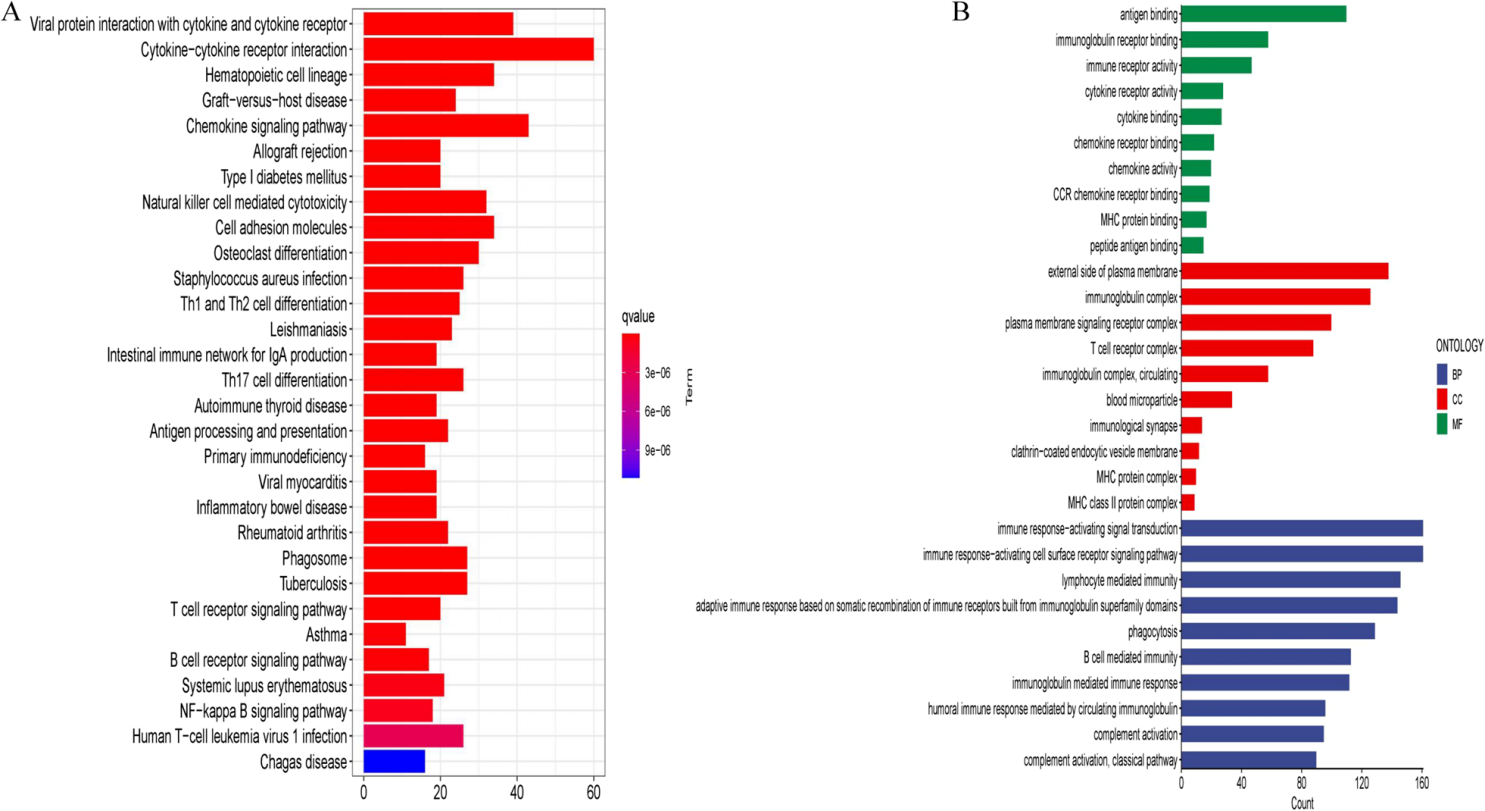
Exploration of the enrichment difference between the two risk groups. **A** Difference of KEGG pathway between the two risk groups. **B** Difference of GO analysis between the two risk groups

The potential mechanism of the DEGs between the high and low-risk groups was explored by KEGG pathway analysis. KEGG analysis showed that the DEGs were mainly enriched in viral protein interaction with cytokine and cytokine receptor, cytokine-cytokine receptor interaction, chemokine signaling pathway, natural killer cell-mediated cytotoxicity, and cell adhesion molecules (Fig. 7A).

We performed a GO analysis to determine the biological characteristics of the DEGs between the two groups. BP analysis showed that the biological processes were significantly enriched in antigen binding, immunoglobulin receptor binding, cytokine receptor activity, cytokine receptor activity, and cytokine binding. CC analysis showed that the DEGs functioned in the external side of the plasma membrane, immunoglobulin complex, plasma membrane signaling receptor complex, T cell receptor complex, and circulating immunocomplex. MF analysis showed that the DEGs were mainly enriched in antigen binding, immunoglobulin receptor binding, immune receptor activity, cytokine receptor activity, and cytokine binding (Fig. 7B).

### Immune function and TMB difference between the high- and low-risk groups and the response to immunotherapy

**Fig. 8:**
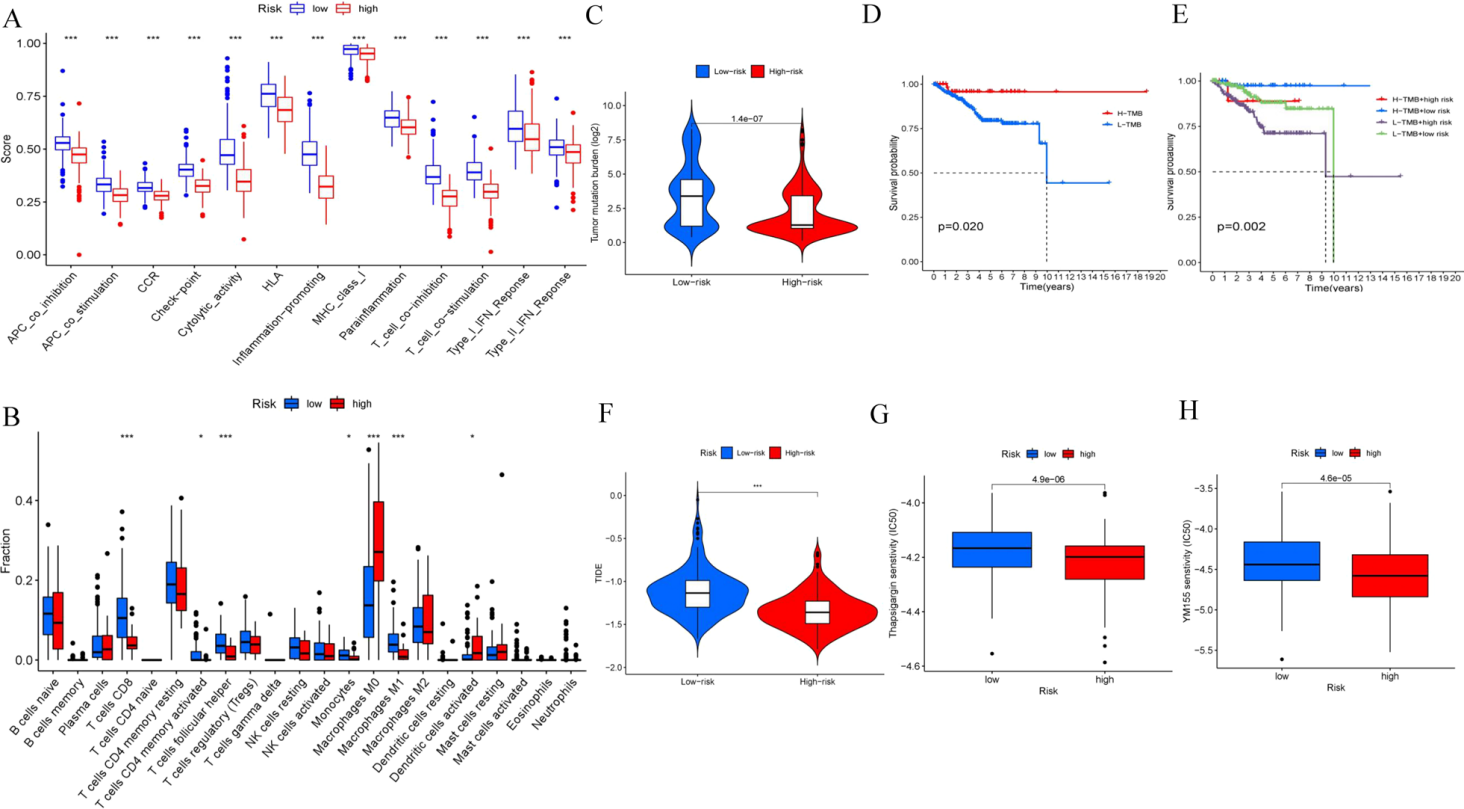
Exploration of the immune functions, TMB difference, immunotherapy and chemotherapy sensitivity between the two risk groups. **A** The difference in immune functions. **B** The difference in immune cells fractions. **C** The difference in tumor mutation burden. **D** The survival probability of the high-TMB group was better than the low-TMB group. **E** The survival probability of the TMB group combined with different risks. The group with the high TMB and the low risk score had the best prognosis, and the group with the low TMB and the high risk score had the worst prognosis. **F** The TIDE score of the low-risk group was higher than that of the high-risk group, indicating that the immunotherapy effect in the low-risk group was worse than that in the high-risk group. **G–H** IC50 Values of YM155 and Thapsigargin were higher in the low-risk group

We found that the low-risk group had better immune functions in APC coinhibition, APC costimulation, CCR, checkpoint, cytolytic activity, HLA, inflammation-promoting, MHC class I, parainflammation, T cell coinhibition, T cell costimulation, type I interferon response, and type II interferon response (Fig. 8A).

CD8 T cells, activated CD4 memory T cells, follicular helper T cells, monocytes, and M1 macrophages in the low-risk group were more than in the high-risk group. M0 macrophage and activated dendritic cells in the high-risk group were more than in the low-risk group (Fig. 8B).

TMB was significantly higher in the low-risk group than in the high-risk group (*P* < 0.001) (Fig. 8C). Kaplan–Meier analysis was used to evaluate the prognosis of endometrial adenocarcinoma according to TMB combined with the risk score. The results showed that TMB (Fig. 8D, *P* = 0.02) was significantly positively associated with prognosis. Patients in the high-TMB group had a better prognosis than the low-TMB group, no matter with a high risk or a low risk. High-TMB patients with low-risk scores had the best prognosis (Fig. 8E, *P* < 0.001). In addition, the TIDE score was calculated by the TIDE online tool to investigate the effectiveness of immune checkpoint inhibitors in the two groups. The TIDE score of the low-risk group was higher than that of the high-risk group, indicating that the immunotherapy effect in the low-risk group was worse than that in the high-risk group (Fig. 8F, *P* < 0.001).

### Response of high- and low-risk patients to chemotherapy

According to the “pRRophetic" algorithm, we predicted the IC50 values of 56 chemotherapeutic drugs relevant to risk score, including 5-Fluorouracil, AC220, AP-24534, AS605240, AUY922, BAY 61-3606, Bexarotene, Bleomycin, CAL-101, CGP-60474, CGP-082996, CP466722, Crizotinib, Cyclopamine, DMOG, FMK, FR-180204, FTI-277, Genentech Cpd 10, GSK429286A, HG-6-64-1, IPA-3, JQ12, JW-7-24-1, KIN001-102, LAQ824, LFM-A13, LY317615, Midostaurin, NG-25, PAC-1, Paclitaxel, Pazopanib, PHA-665752, Phenformin, Rapamycin, Roscovitine, Ruxolitinib, Salubrinal, STF-62247, Sunitinib, TGX221, Thapsigargin, Tipifarnib, TL-1-85, TL-2-105, Tubastatin A, WH-4-023, WZ-1-84, XL-184, XMD8-85, XMD14-99, YM155, Zibotentan, Z-LLNle-CHO, and ZSTK474 (Additional files 3, 4, 5: Figs. S1–S3). By comparing the IC50 values of these associated drugs in the two-risk group, the values of YM155 and Thapsigargin were higher in the low-risk group, and the values of the other drugs were higher in the high-risk group (Wilcoxon test, *P* < 0.001; Fig. 8G–H, Additional files 6, 7, 8: Figs. S4–S6).

### Development and validation of a prognostic nomogram

**Fig. 9:**
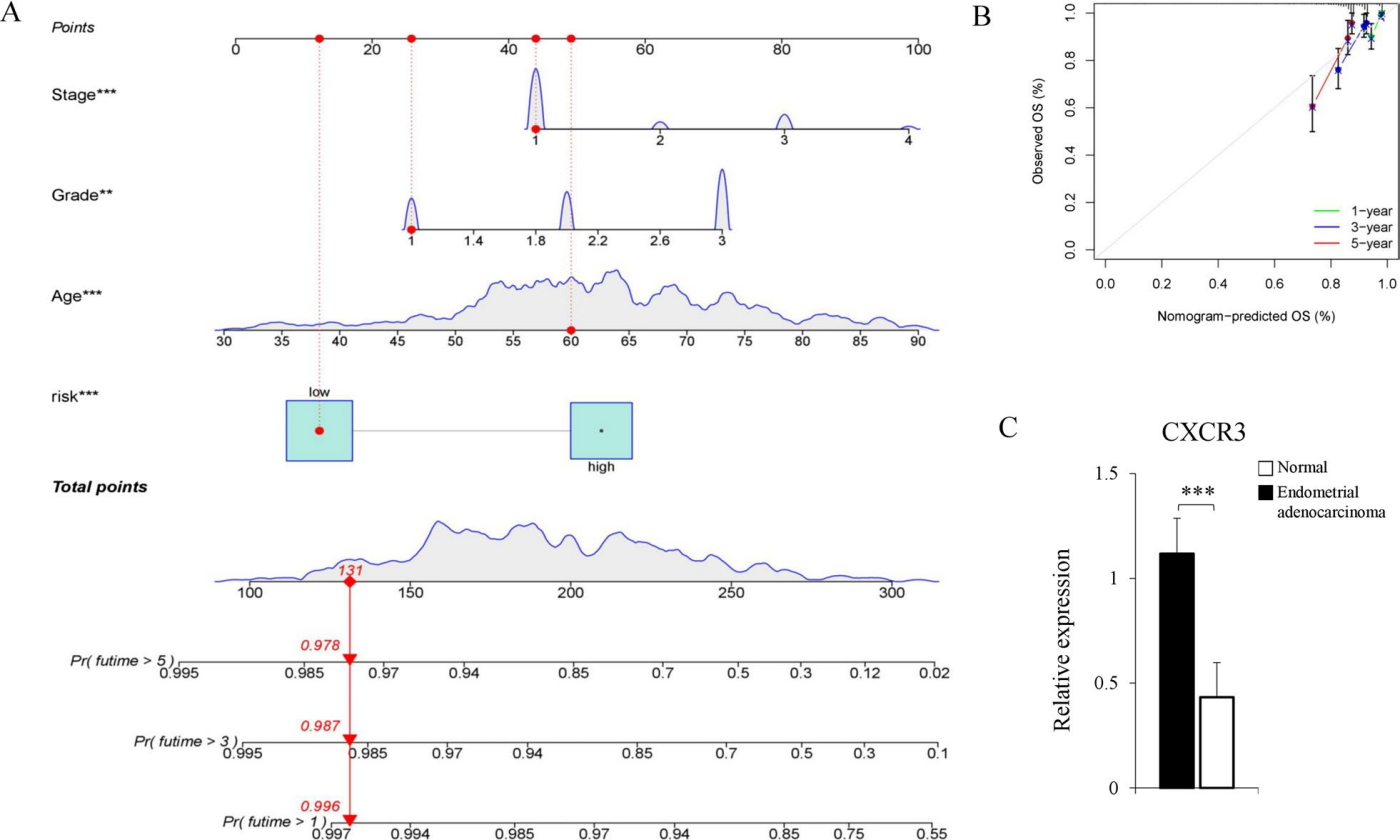
Development and validation of a new predictive nomogram and qPCR verification. **A** The predictive nomogram was used to predict the survival probability at one year, three years, and five years). **B** Calibration curves of the nomograms at one year, three years, and five years. The X-axis represents the predicted probability, and the Y-axis represents the actual probability. The error line represents a 95% confidence interval. 45° represents a perfect prediction and the excellent performance of our nomogram. **C** The qPCR verification of the relative expression of CXCR3 between the normal endometrium and the endometrial adenocarcinoma patients (t-test, *P* < 0.001)

Considering the prognostic significance of the gene biomarker, we tried to combine it with clinical factors to better predict the survival of patients with endometrial adenocarcinoma. First, age, histological grade, FIGO stage, and risk group were included to develop the predictive nomogram of endometrial adenocarcinoma. Total points were calculated by summing up each item in the nomogram and predicting the survival rate of one year, three years, and five years (Fig. 9A). Second, the 1-year, 3-year, and 5-year calibration curves indicated high consistency between the predicted and actual survival ratios (Fig. 9B).

### The relative expression of CXCR3 in endometrial adenocarcinoma

The expression of CXCR3 was tested by qPCR with clinical samples from adenocarcinoma patients and normal endometrium from patients with other diseases that need to remove the uterus to identify the effect of CXCR3 (Fig. 9C), indicating that the expression of CXCR3 was lower in endometrial adenocarcinoma.

## Discussion

In our study, we developed and validated a gene biomarker and prognostic nomogram model combining gene markers and clinical factors (age, histological grade, and FIGO stage) for patients with endometrial adenocarcinoma, which had not been reported before. In endometrial adenocarcinoma, CXCR3 was identified as a meaningful anti-tumor gene. The Nomogram model can effectively predict 1-year, 3-year, and 5-year survival ratios.

CXCR3 is a G protein-coupled receptor that binds to ELR-negative CXC chemokines, which could affect immune responses [65] and be required for the efficacy of anti-PD-1 therapy [66]. CXCR3 consists of CXCR3-A, CXCR3-B, and CXCR3-alt. The first two have opposite physiological functions. CXCR3-A mainly exists in hematopoietic cells, which could promote tumor progression by survival, cell proliferation, chemotaxis, invasion, and metastasis [65]. CXCR3-B mainly exists in epithelial cells, which could lead to growth inhibition, apoptosis, and anti-angiogenesis [65]. Higher CXCR3 expression has been reported to be related to a good prognosis of renal and gastric cancer [67–69]. However, there were also some studies indicating that higher CXCR3 expression predicted poor survival in solid tumor l [70].

CXCR3 expressed on regulatory T cells could induce peripheral CD4 T cells to differentiate into regulatory T cells and improves effector T cell function [71], which is consistent with the higher fraction of CD4+ T cells and CD8+ T cells in the low-risk group in our study. Chemokine ligands 9, 10, 11 (CXCL 9-11) interacting with CXCR3 expressed on monocytes, T cells, and NK cells may be involved in inhibiting angiogenesis [72], which is consistent with our study that high expression of CXCR3 had better survival outcome and the fraction of CD4+ T cells, CD8+ T cells and monocytes were higher in the low-risk group. CXCL11 could inhibit angiogenesis, affect the proliferation of different cell types, increase adhesion properties, inhibit M2 macrophage polarization and promote the migration of some immune cells [73], which was consistent with the higher fraction of M1 macrophage in the low-risk group. However, CXCL9/CXCR3 axis has been reported to activate Akt signaling pathway, accompany EMT and cytoskeleton rearrangement, and promote invasion and metastasis in tongue squamous cell carcinoma [74]. CXCL10 promotes CXCR3 expressing cancer cells transported to bone [75]. These different functions of CXCR3 may be attributed to its different variant types and different cell types.

Nevertheless, in our study, the qPCR result showed that the relative expression of CXCR3 in endometrial adenocarcinoma was lower than in normal endometrium. This result indicated that CXCR3 in endometria adenocarcinoma might be an anti-tumor effect. T-follicular helper cells (TFH) may contribute to the treatment of endometrial cancer, and high TFH infiltration shows clinical application potential in anti-PD-1 treatment [76]. The increase in the number of CD8+ T cells at the invasive boundary may improve the survival of patients with endometrial cancer [77]. In our study, the follicular T cells and CD8+ T cells were all significantly increased in the low-risk group, which had a higher anti-tumor effect.

In addition, we revealed the immune function, including APC coinhibition, APC costimulation, CCR, checkpoint, cytolytic activity, HLA, inflammation-promoting, MHC-I, parainflammation, T cell coinhibition, T cell costimulation, type I-IFN-response, and type II-IFN-response were better in the low-risk group. By KEGG pathway and GO analysis based on the DEGs between the two risk groups, we found that viral protein interaction with cytokine and cytokine receptor, cytokine-cytokine receptor interaction, chemokine signaling pathway, natural killer cell-mediated cytotoxicity and cell adhesion molecules were enriched obviously. The most critical immune-related pathway in the low-risk group was the cytokine-cytokine receptor pathway, which played an important role in B cell-related immune diseases, such as autoimmune diseases, and malignant diseases including, lymphoma, leukemia [78], colorectal cancer [79], renal cell carcinoma [80] hepatocellular carcinoma [81], non-squamous non-small cell lung cancer [82], lung adenocarcinoma [83], and AIDS-related Kaposi sarcoma (AIDS-KS) [84]. TMB could be a candidate biomarker to estimate the possible response to immunotherapy [85, 86]. Zhou [87] reported that the survival of the low-TMB group was worse than the high-TMB group, which was consistent with our study. High TMB might possess a good prognosis for PD-1/PD-L1 blockade in diverse tumors [86]. High-TMB tumors with microsatellite stability can better respond to pembrolizumab with longer PFS than low/intermediate TMB tumors [88]. All these findings contribute to developing new therapeutic strategies for endometrial adenocarcinoma and provide an opportunity for the further immune exploration of endometrial adenocarcinoma.

Currently, there already exist some different immune-related signatures. For example, Chen [89] found that immune and stromal scores had a relationship with the prognosis of EC patients by the “ESTIMATE” R tool. Based on the immune and stromal scores and the intersected differentially expressed genes, eight immune-related genes (AQP4, ARHGAP36, CACNA2D2, CTSW, NOL4, SIGLEC1, TMEM150B, and TRPM5) were then identified by LASSO algorithm and Random-forest algorithm. Tang [90]used the Spearman correlation analysis to identify immune-related pseudogenes and then developed a risk signature consisting of nine immune-related pseudogenes by univariate Cox regression, LASSO, and multivariate to predict the prognosis. Meng [91] used the conjoint Cox regression model to develop a signature consisting of seven immune-related genes (CBLC, PLA2G2A, TNF, NR3C1, APOD, TNFRSF18, and LTB). They all indicated that patients in the low-risk group had a significantly longer survival time than those in the high-risk group. However, analyzed by different bioinformatic tolls, the signatures were identified differently and consisted of multiple genes leading to needing to be more convenient for helping clinical practice.

However, first, our study identified CXCR3 as the solely optimized immune-related prognostic biomarker. Although it was not newly found, it was first used as a predictive gene marker in adenocarcinoma. As CXCR3 has been studied for years, its effect on cancer is more well-known and convenient for clinical applications. Moreover, it had been developed and validated in the TCGA cohort, which had many cases to guarantee its reliability and stability. Second, we revealed the potential immune pathways, immune cells, tumor mutation burden, and TIDE scores between the low-risk and the high-risk group, which might help find effective immunotherapy. Third, we establish a nomogram to predict the prognosis. The last but most crucial finding was the discovery of sensitive drugs in the high-risk and low-risk groups. The high-risk group was more sensitive to YM155 and Thapsigargin, and the low-risk group was more sensitive to the other 54 drugs by comparing the IC50 values of the 56 sensitive drugs between the two groups [92].

This study also has some limitations and deserves further investigation. First, no suitable external cohort with clinical data can be used to demonstrate the model further. Second, more in vitro and in vivo studies are needed to explore the physiological mechanisms. Critical immune cells, including M1 macrophages, memory-activated CD4+ T cells, CD8+ T cells, and follicular helper T cells, have been proven beneficial to the survival of endometrial adenocarcinoma patients, and the exact underlying mechanism needs to be further studied.

In conclusion, we successfully developed and validated a simple gene biomarker and a new nomogram prognosis model based on exploiting the data from TCGA and GEO databases with bioinformatics tools. Our study also revealed some interesting immune cells and pathways in endometrial adenocarcinoma, which can be used as potential immunotherapeutic targets.

## Supplementary Information

**Additional file 1**: **Table S1**. Identification of overlapping genes and estimate scores.

**Additional file 2**: **Table S2**. Identification and validation of the prognostic immune-gene biomarker

**Additional file 3**: **Fig. S1**. Screening the sensitive drugs relevant to the risk scores.

**Additional file 4**: **Fig. S2**. Screening the sensitive drugs relevant to the risk scores.

**Additional file 5**: **Fig. S3**. Screening the sensitive drugs relevant to the risk scores.

**Additional file 6**: **Fig. S4**. Comparing the two risk groups' IC50 values of the relevantly sensitive drugs.

**Additional file 7**: **Fig. S5**. Comparing the two risk groups' IC50 values of the relevantly sensitive drugs.

**Additional file 8**: **Fig. S6**. Comparing the two risk groups' IC50 values of the relevantly sensitive drugs.

## Data Availability

The datasets analyzed during this study are available in the TCGA repository (https://portal.gdc.cancer.gov/) and the GEO database (https://www.ncbi.nlm.nih.gov/geo/query/acc.cgi?acc=gse17025).
