# Supplementary figures and images for: CXCR3 predicts the prognosis of endometrial adenocarcinoma
Source: BMC Med Genomics. 2023 Feb 7;16:20. doi: 10.1186/s12920-023-01451-9 (PMC9903462; doi:10.1186/s12920-023-01451-9)

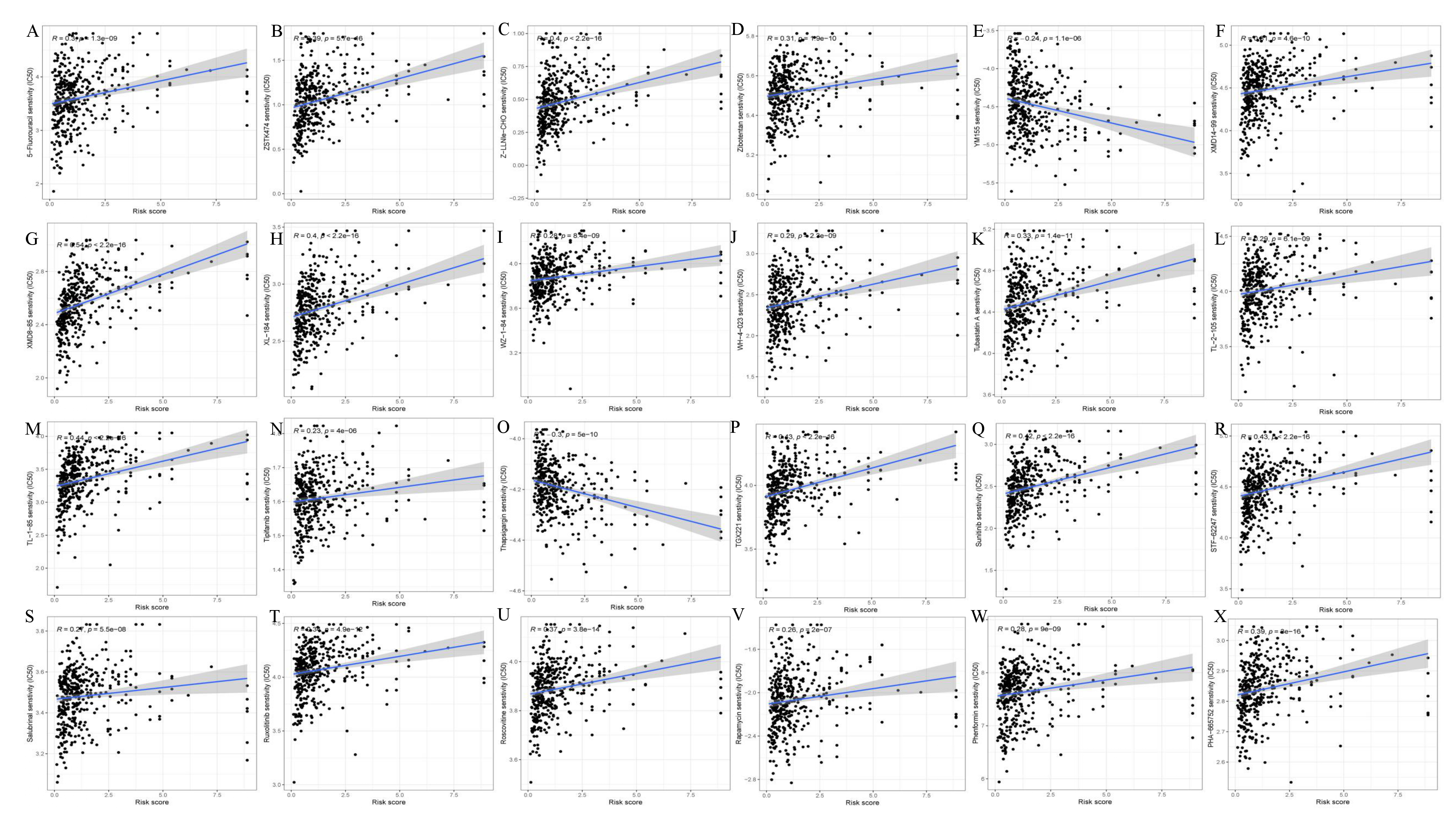

Supplement: Supplementary file 3 — Additional file 3: Fig. S1. Screening the sensitive drugs relevant to the risk scores. [file 12920_2023_1451_MOESM3_ESM.tif]

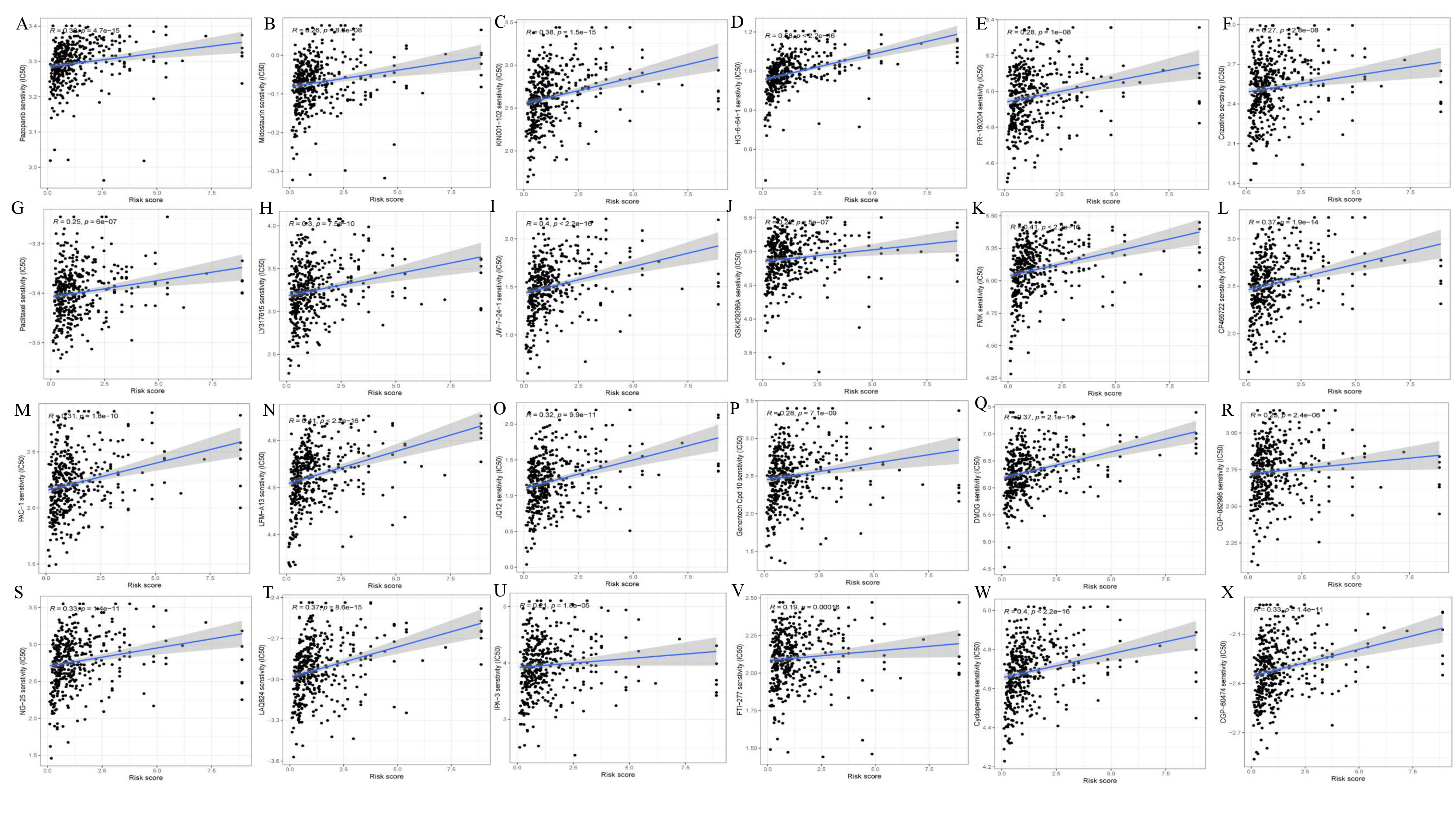

Supplement: Supplementary file 4 — Additional file 4: Fig. S2. Screening the sensitive drugs relevant to the risk scores. [file 12920_2023_1451_MOESM4_ESM.tif]

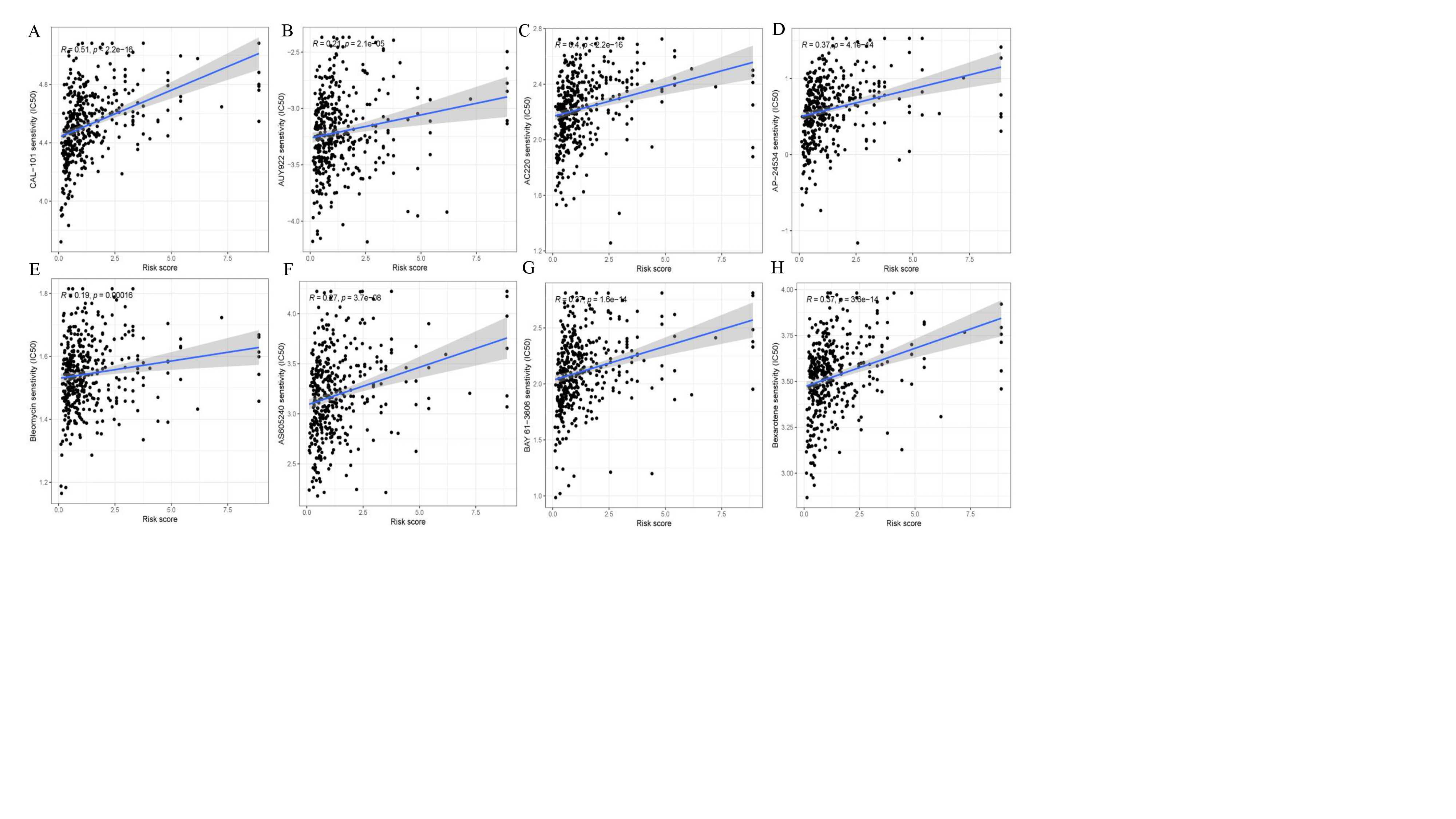

Supplement: Supplementary file 5 — Additional file 5: Fig. S3. Screening the sensitive drugs relevant to the risk scores. [file 12920_2023_1451_MOESM5_ESM.tif]

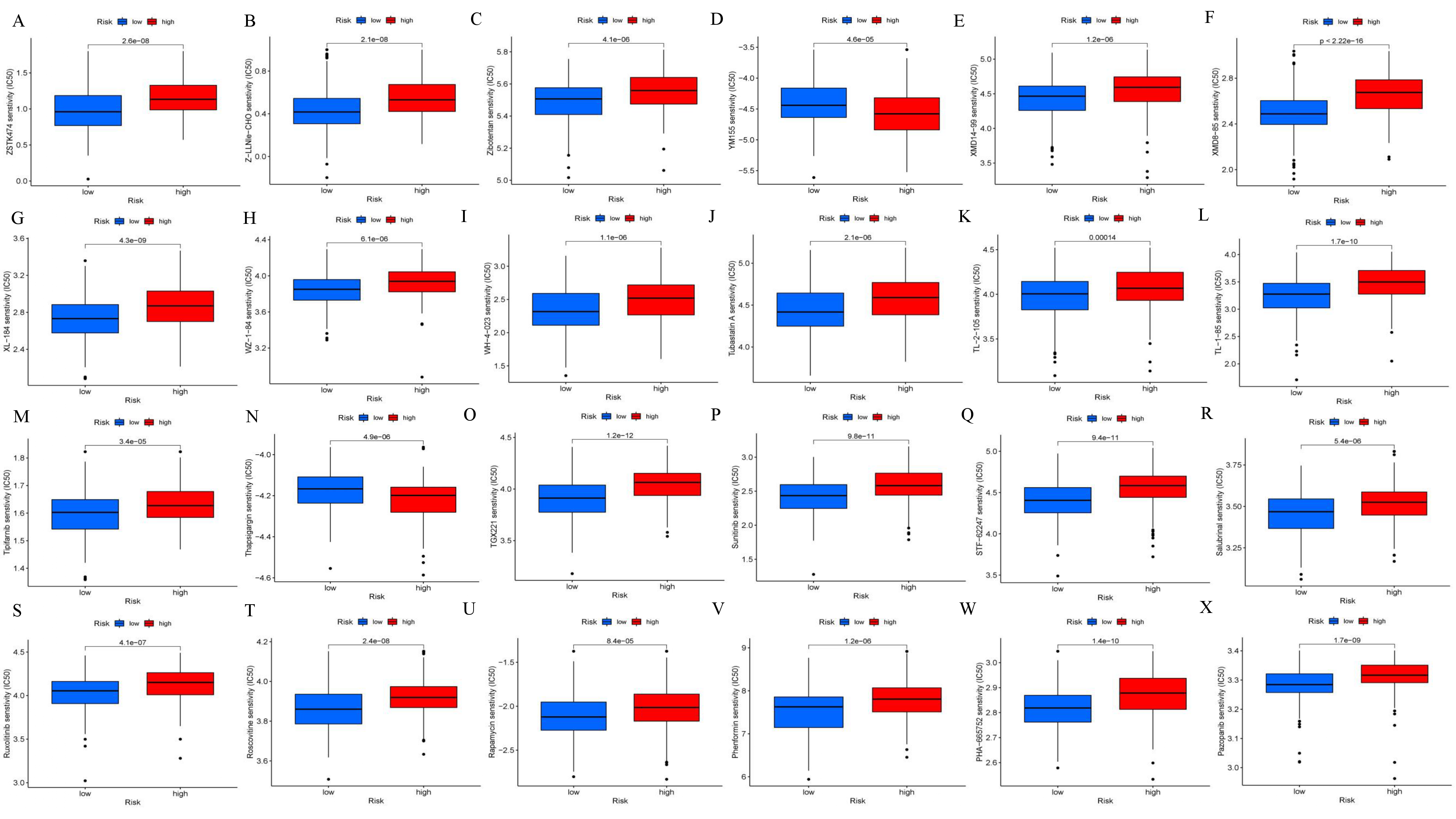

Supplement: Supplementary file 6 — Additional file 6: Fig. S4. Comparing the two risk groups' IC50 values of the relevantly sensitive drugs. [file 12920_2023_1451_MOESM6_ESM.tif]

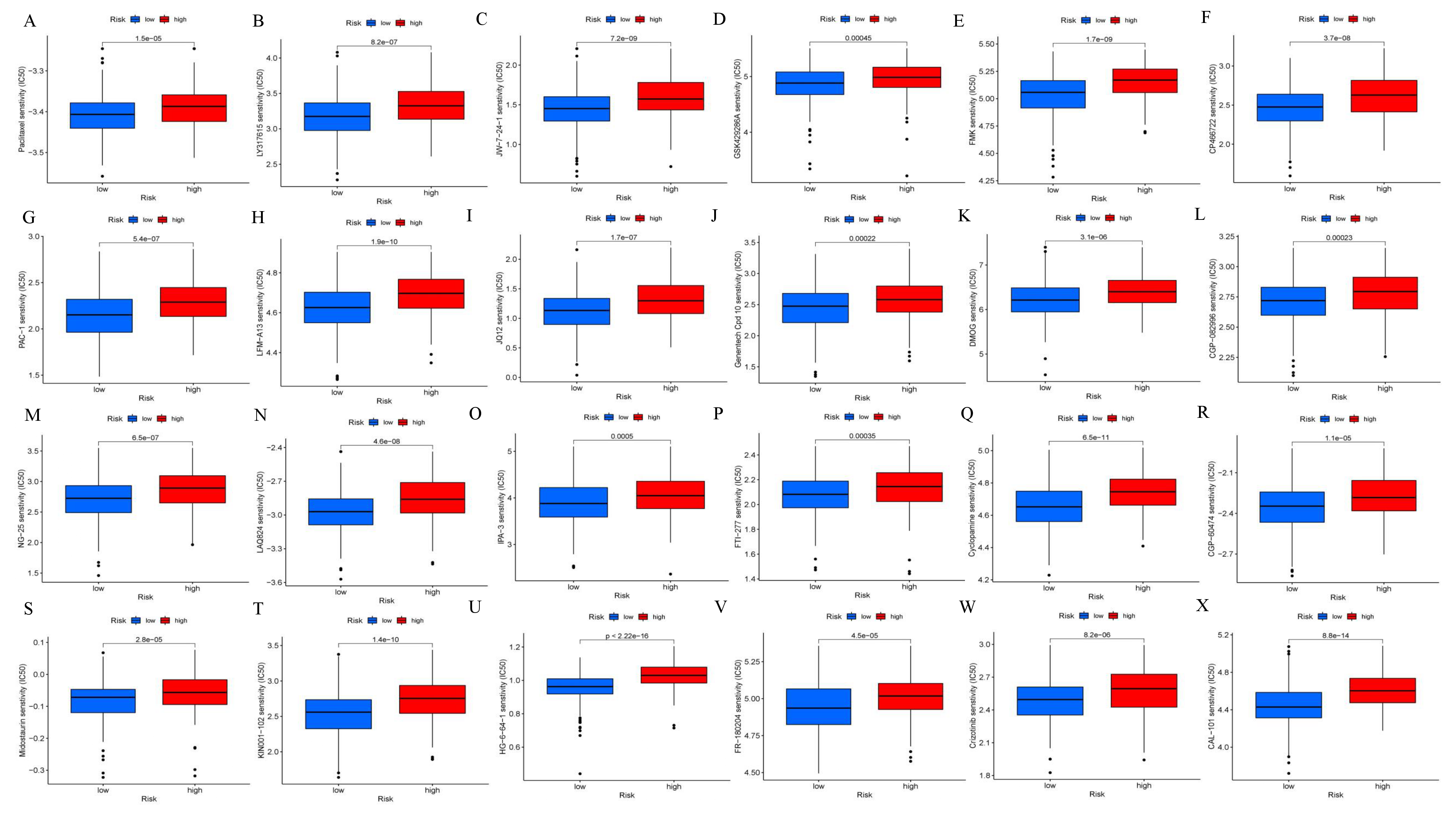

Supplement: Supplementary file 7 — Additional file 7: Fig. S5. Comparing the two risk groups' IC50 values of the relevantly sensitive drugs. [file 12920_2023_1451_MOESM7_ESM.tif]

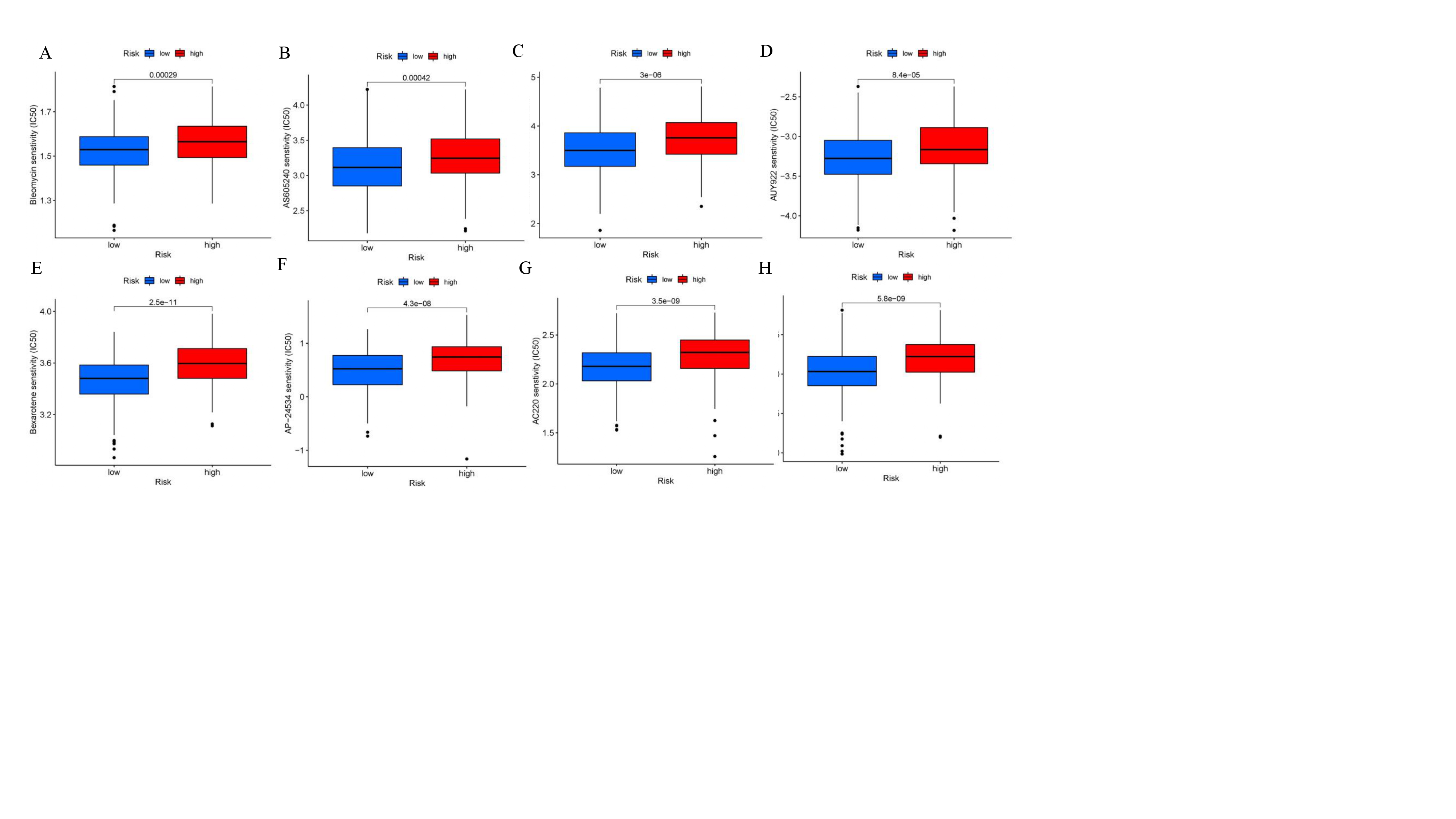

Supplement: Supplementary file 8 — Additional file 8: Fig. S6. Comparing the two risk groups' IC50 values of the relevantly sensitive drugs. [file 12920_2023_1451_MOESM8_ESM.tif]
